# Supplementary material for: Anti-SARS-CoV-2 Antibody Status at the Time of Hospital Admission and the Prognosis of Patients with COVID-19: A Prospective Observational Study
Source: Infect Dis Rep. 2022 Dec 11;14(6):1004–16. doi: 10.3390/idr14060100 (PMC9779184; doi:10.3390/idr14060100)
Supplement: Supplementary file 1 [file idr-14-00100-s001.zip › idr-2035341-supplementary.pdf]

Supplementary Table S1. Kolmogorov-Smirnov test for normal distribution of quantitative variables obtained at the time of admission in the characteristics of the cohort of patients with positive anti-S-SARS-CoV-2 immunoglobulins and cohort of patients with negative anti-S-SARS-CoV-2 immunoglobulins.

	**anti-SARS-CoV-2 Ig positive p-value (KS – test)**	**anti-SARS-CoV-2 Ig Negative p-value (KS – test)**
Age (years)	0.268	0.246
BMI (kg.m^{-2})	0.0001	0.005
CRP (mg/L)	0.024	0.012
IL-6 (pg/mL)	0.0001	0.0001
N/L ratio	0.0001	0.0001
GFR (ml/min)	0.015	0.265
D-dimer (mg/L)	0.0001	0.0001
Duration of symptoms to admission (days)	0.0001	0.0001
Hospitalisation length (days)	0.0001	0.0001
Length of follow up (days)	0.0001	0.0001
Survival in deceased patients (Days)	0.0001	0.0001

BMI: body mass index; CRP: C-reactive protein; GFR: glomerular filtration rate; IL-6: interleukin 6; n: number, KS - test: Kolmogorov-Smirnov test; N/L ratio: neutrophil-to-lymphocyte ratio; p-value: probability

Supplementary Table S2: Binary logistic regression model – forward stepwise method of association of in-hospital death with anti-S SARS-CoV-2 negativity and other variables. Variables in the equation of regression model.

		Beta	Beta SE	Wald	p-value	OR	Lower 95% CI	Upper 95% CI
Step 1	Comorbidity number (n)	0.723	0.136	28.326	0.000	2.061	1.579	2.689
	Constant	-2.890	0.255	128.897	0.000	0.056		
Step 2	Moderate disease			20.893	0.000			
	Severe disease	-21.212	5007.119	0.000	0.997	0.000	0.000	
	Critical disease	-2.240	0.490	20.893	0.000	9.433	3,600	24.392
	Comorbidity number (n)	0.759	0.146	26.877	0.000	2.136	1.603	2.846
	Constant	-.768	.472	2.652	.103	.464		
Step 3	Moderate disease			13.190	0.001			
	Severe disease	-20.850	4906.200	0.000	0.997	0.000	0.000	
	Critical disease	-1.877	0.517	13.190	0.000	6.536	2.381	17.857
	Comorbidity number (n)	0.756	0.149	25.844	0.000	2.130	1.592	2.852
	Anti-S SARS-CoV-2 negative	1.242	0.347	12.776	0.000	3.461	1.752	6.837
	Constant	-1.751	0.577	9.219	0.002	0.174		
Step 4	Moderate disease			13.170	0.001			
	Severe disease	-21.081	4775.218	0.000	0.996	0.000	0.000	
	Critical disease	-1.954	0.538	13.170	0.0001	7.042	2.457	20.408
	Comorbidity number (n)	0.483	0.164	8.620	0.003	1.621	1.174	2.237
	Age (years)	0.056	0.017	10.942	0.001	1.058	1.023	1.093
	Anti-S SARS-CoV-2 negative	1.205	0.353	11.671	0.001	3.336	1.671	6.659
	Constant	-4.899	1.142	18.393	0.000	0.007		
Step 5	Moderate disease			12.694	0.002			
	Severe disease	-21.120	4748.158	0.000	0.996	0.000	0.000	
	Critical disease	-1.957	0.549	12.694	0.0001	7.460	2.475	22.222
	D-dimer (mg/l)	0.092	0.046	4.037	0.045	1.096	1.002	1.199
	Comorbidity number (n)	0.496	0.166	8.956	0.003	1.641	1.186	2.271
	Age (years)	0.053	0.017	9.669	0.002	1.055	1.020	1.091
	Anti-S SARS-CoV-2 negative	1.240	0.357	12.066	0.001	3.457	1.717	6.961
	Constant	-4.923	1.151	18.281	0.000	0.007		

A forward stepwise method (probability for stepwise: entry $p < 0.05$) for binary logistic regression has been used to reduce the number of independent variables entering the model to reduce the probability of model overfitting. CI: confidence interval; n: number; OR: odds ratio; p-value: probability (multivariate), SE: standard error.

Supplementary Table S3: Binary logistic regression model – forward stepwise method of association of in-hospital death with anti-S SARS-CoV-2 negativity and other variables. Variables not in the equation of regression model.

		Score	p-value
Step 1	Age (years)	11.282	0.001
	Duration of symptoms (days)	0.684	0.408
	Baricitinib	3.821	0.051
	Anti-S SARS-CoV-2 negative	20.893	0.000
	BMI (kg.m^{-2})	0.067	0.795
	CRP (mg/L)	6.002	0.014
	IL-6 (pg/mL)	2.566	0.109
	N/L ratio	6.800	0.009
	D-dimer (mg/L)	3.430	0.064
	Severity	26.007	0.000
Step 2	Age (years)	14.200	0.000
	Duration of symptoms (days)	1.440	0.230
	Baricitinib	0.686	0.407
	Anti-S SARS-CoV-2 negative	14.200	0.000
	BMI (kg.m^{-2})	0.006	0.938
	CRP (mg/L)	2.392	0.122
	IL-6 (pg/mL)	1.108	0.293
	N/L ratio	5.882	0.015
	D-dimer (mg/L)	5.513	0.019
Step 3	Age	13.024	0.000
	Duration of symptoms (days)	2.218	0.136
	Baricitinib	0.083	0.774
	BMI (kg.m^{-2})	0.133	0.715
	CRP (mg/L)	4.827	0.028
	IL-6 (pg/mL)	2.040	0.153
	N/L ratio	7.201	0.007
	D-dimer (mg/L)	6.088	0.014
Step 4	Duration of symptoms (days)	1.617	0.204
	Baricitinib	0.334	0.563
	BMI (kg.m^{-2})	0.522	0.470
	CRP (mg/L)	4.118	0.042
	IL-6 (pg/mL)	1.456	0.228
	N/L-ratio	4.003	0.045
	D-dimer (mg/L)	4.942	0.026
Step 5	Duration of symptoms (days)	1.598	0.206
	Baricitinib	0.450	0.502
	BMI (kg.m^{-2})	0.554	0.457
	CRP (mg/L)	2.840	0.092
	IL-6 (pg/mL)	0.912	0.339
	N/L ratio	3.507	0.061

BMI: body mass index; CRP: C-reactive protein; IL-6: interleukin 6; n: number, N/L ratio: neutrophil-to-lymphocyte ratio; OR: odds ratio; p-value: probability (univariate)

Supplementary Table S4: Binary logistic regression model – forward stepwise method of association of of in-hospital death with anti-S SARS-CoV-2 IgM negativity and other variables. Variables in the equation of regression model.

		Beta	Beta SE	Wald	p-value	OR	Lower 95% CI	Upper 95% CI
Step 1	Comorbidity number (n)	0.752	0.138	29.845	0.000	2.121	1.619	2.777
	Constant	-2.936	0.260	127.826	0.000	0.053		
Step 2	Moderate disease			21.126	0.000			
	Severe disease	-21.228	5030.084	0.000	0.997	0.000	0.000	
	Critical disease	-2.269	0.494	21.126	0.000	9.708	3.676	25.64
	Comorbidity number (n)	0.794	0.149	28.421	0.000	2.212	1.652	2.962
	Constant	-0.802	0.474	2.862	0.091	0.449		
Step 3	Moderate disease			12.831	0.002			
	Severe disease	-20.849	4921.019	0.000	0.997	0.000	0.000	
	Critical disease	-1.873	0.523	12.831	0.0001	6.493	2.336	18.182
	Anti-S SARS-CoV-2 IgM negative	1.396	0.361	14.992	0.0001	4.041	1.993	8.194
	Comorbidity number (n)	0.777	0.151	26.376	0.0001	2.176	1.617	2.927
	Constant	-1.900	0.588	10.432	0.001	0.150		
Step 4	Age (years)	0.062	0.018	12.465	0.0001	1.064	1.028	1.101
	Moderate disease			13.039	0.001			
	Severe disease	-21.119	4769.362	0.000	0.996	0.000	0.000	
	Critical disease	-1.983	0.549	13.039	0.0001	7.246	2.475	21.277
	Anti-S SARS-CoV-2 IgM negative	1.335	0.366	13.277	0.0001	3.801	1.853	7.795
	Comorbidity number (n)	0.483	0.168	8.324	0.004	1.622	1.168	2.252
	Constant	-5.370	1.179	20.761	0.0001	0.005		
Step 5	Age (years)	0.059	0.018	11.171	0.001	1.061	1.025	1.098
	Moderate disease			12.623	0.002			
	Severe disease	-21.165	4740.599	0.000	0.996	0.000	0.000	
	Critical disease	-1.990	0.560	12.623	0.0001	7.299	2.439	21.739
	Anti-S SARS-CoV-2 IgM negative	1.385	0.372	13.846	0.0001	3.994	1.926	8.284
	D-dimer (mg/L)	0.096	0.046	4.227	0.040	1.100	1.004	1.205
	Comorbidity number (n)	0.495	0.169	8.612	0.003	1.641	1.179	2.283
	Constant	-5.412	1.189	20.724	0.0001	0.004		
Step 6	Age (years)	0.055	0.018	9.306	0.002	1.056	1.020	1.094
	Moderate disease			11.703	0.003			
	Severe disease	-21.077	4700.488	0.000	0.996	0.000	0.000	
	Critical disease	-1.909	0.558	11.703	0.001	6.757	2.257	20.000
	Anti-S SARS-CoV-2 IgM negative	1.513	0.387	15.261	0.0001	4.542	2.126	9.706
	N/L-ratio	0.042	0.021	4.034	0.045	1.043	1.001	1.086
	D-dimer (mg/L)	0.092	0.047	3.815	0.051	1.096	1.000	1.202

	Comorbidity number (n)	0.500	0.171	8.555	0.003	1.649	1.179	2.305
	Constant	-5.653	1.219	21.489	0.0001	0.004		

A forward stepwise method (probability for stepwise: entry $p < 0.05$) for binary logistic regression been used to reduce the number of independent variables entering the model to reduce the probability of model overfitting. CI: confidence interval; n: number, OR: odds ratio; , N/L ratio: neutrophil-to-lymphocyte ratio; p-value: probability (multivariate), SE: standard error.

Supplementary Table S5: Binary logistic regression model – forward stepwise method of association of in-hospital death with anti-S SARS-CoV-2 IgM negativity and other variables. Variables not in the equation of regression model.

		Score	p-value
Step 1	Age (years)	12.783	0.0001
	Duration of symptoms (days)	0.694	0.405
	Moderate disease	38.585	0.0001
	Severe disease	8.154	0.004
	Critical disease	0.871	0.351
	Anti-S SARS-CoV-2 IgM negative	23.194	0.000
	BMI (kg.m^{-2})	0.082	0.774
	IL-6 (pg/mL)	2.549	0.110
	CRP (mg/L)	6.043	0.014
	N/L ratio	5.891	0.015
	D-dimer (mg/L)	9.502	0.002
	Baricitinib	3.482	0.062
Step 2	Age (years)	15.059	0.0001
	Duration of symptoms (days)	1.431	0.232
	Anti-S SARS-CoV-2 IgM negative	16.508	0.0001
	BMI (kg.m^{-2})	0.009	0.925
	IL-6 (pg/mL)	1.253	0.263
	CRP (mg/L)	2.232	0.135
	N/L ratio	4.929	0.026
	D-dimer (mg/L)	5.674	0.017
	Baricitinib	0.596	0.440
Step 3	Age (years)	13.128	0.0001
	Duration of symptoms (days)	2.212	0.137
	BMI (kg.m^{-2})	0.255	0.614
	IL-6 (pg/mL)	2.623	0.105
	CRP (mg/L)	5.042	0.025
	N/L ratio	7.605	0.006
	D-dimer (mg/L)	5.842	0.016
	Baricitinib	0.110	0.741
Step 4	Duration of symptoms (days)	1.572	0.210
	BMI (kg.m^{-2})	0.264	0.607
	IL-6 (pg/mL)	2.202	0.138
	CRP (mg/L)	4.231	0.040
	N/L ratio	4.340	0.037
	D-dimer (mg/L)	4.369	0.037
	Baricitinib	0.454	0.501
Step 5	Duration of symptoms (days)	1.531	0.216
	BMI (kg.m^{-2})	0.256	0.613

	IL-6 (pg/mL)	2.073	0.150
	CRP (mg/L)	2.835	0.092
	N/L ratio	4.002	0.045
	Baricitinib	0.667	0.414
Step 6	Duration of symptoms (days)	1.507	0.220
	BMI (kg.m^{-2})	0.383	0.536
	IL-6 (pg/mL)	1.576	0.209
	CRP (mg/L)	0.643	0.423
	Baricitinib	0.764	0.382

BMI: body mass index; CRP: C-reactive protein; IL-6: interleukin 6; n: number, N/L ratio: neutrophil-to-lymphocyte ratio; p-value: probability (univariate)

Supplementary Table S6: Binary logistic regression model – forward stepwise method of association of of in-hospital death with anti-S SARS-CoV-2 IgG negativity and other variables. Variables in the equation of regression model.

		Beta	Beta SE	Wald	p-vaue	OR	Lower 95% CI	Upper 95% CI
Step 1	Comorbidity number (n)	0.752	0.138	29.845	0.0001	2.121	1.619	2.777
	Constant	-2.936	0.260	127.826	0.0001	0.053		
Step 2	Moderate disease			21.126	0.0001			
	Severe disease	-21.228	5030.084	0.000	0.997	0.000	0.000	
	Critical disease	-2.269	0.494	21.126	0.0001	9,708	3.676	25.641
	Comorbidity number (n)	0.794	0.149	28.421	0.0001	2.212	1.652	2.962
	Constant	-.802	0.474	2.862	0.091	.449		
Step 3	Age (years)	0.066	0.017	14.284	0.0001	1.068	1.032	1.105
	Moderate disease			20.592	0.0001			
	Severe disease	-21.499	4853.950	0.000	0.996	0.000	0.000	
	Critical disease	-2.408	0.531	20.592	0.0001	11.111	3.922	31.250
	Comorbidity number (n)	0.498	0.161	9.509	0.002	1.645	1.199	2.258
	Constant	-4.530	1.126	16.187	0.0001	0.011		
Step 4	Age (years)	0.068	0.018	14.915	0.0001	1.070	1.034	1.108
	Moderate disease			16.709	0.0001			
	Severe disease	-21.326	4770.738	0.000	0.996	0.000	0.000	
	Critical disease	-2.230	0.545	16.709	0.0001	9.259	3.195	27.027
	Comorbidity number (n)	0.492	0.168	8.548	0.003	1.635	1.176	2.273
	Anti-S SARS-CoV-2 IgG negative	1.049	0.382	7.532	0.006	2.855	1.350	6.039
	Constant	-5.509	1.208	20.793	0.0001	0.004		
Step 5	Age (years)	0.065	0.018	13.377	0.0001	1.067	1.031	1.105
	Moderate disease			16.200	0.0001			
	Severe disease	-21.373	4746.051	0.000	0.996	0.000	0.000	
	Critical disease	-2.244	0.558	16.200	0.0001	9.434	3.165	27.778
	Comorbidity number (n)	0.094	0.046	4.142	0.042	1.099	1.003	1.203
	D-dimer (mg/L)	0.505	0.170	8.871	0.003	1.657	1.188	2.310
	Anti-S SARS-CoV-2 IgG negative	1.093	0.389	7.908	0.005	2.983	1.393	6.388
	Constant	-5.527	1.217	20.610	0.0001	0.004		

A forward stepwise method (probability for stepwise: entry $p < 0.05$) for binary logistic regression and has been used to reduce the number of independent variables entering the model to reduce the probability of model overfitting.
CI: confidence interval; n: number; OR: odds ratio; p-value: probability (multivariate), SE: standard error.

Supplementary Table S7: Binary logistic regression model – forward stepwise method of association of in-hospital death with anti-S SARS-CoV-2 IgG negativity and other variables. Variables not in the equation of regression model.

		Score	p-value
Step 1	Age (years)	12.783	0.0001
	Duration of symptoms (days)	0.694	0.405
	Moderate disease	38.585	0.0001
	Severe disease	8.154	0.004
	Critical disease	0.871	0.351

	BMI (kg.m^{-2})	0.082	0.774
	IL6 (ng/l)	2.549	0.110
	CRP (mg/l)	6.043	0.014
	N/L ratio	5.891	0.015
	D-Dimer (mg/l)	9.502	0.002
	Baricitnib	3.482	0.062
	Anti-S SARS-CoV-2 IgG negative	10.427	0.001
Step 2	Age (years)	15.059	0.0001
	Duration of symptoms (days)	1.431	0.232
	BMI (kg.m^{-2})	0.009	0.925
	IL6 (ng/l)	1.253	0.263
	CRP (mg/l)	2.232	0.135
	N/L ratio	4.929	0.026
	D-Dimer (mg/l)	5.674	0.017
	Baricitinib	0.596	0.440
	Anti-S SARS-CoV-2 IgG negative	7.177	0.007
Step 3	Duration of symptoms (days)	0.958	0.328
	BMI (kg.m^{-2})	1.089	0.297
	IL6 (ng/l)	1.097	0.295
	CRP (mg/l)	2.017	0.156
	N/L ratio	2.291	0.130
	D-Dimer (mg/l)	3.402	0.065
	Baricitinib	1.433	0.231
	Anti-S SARS-CoV-2 IgG negative	7.934	0.005
Step 4	Duration of symptoms (days)	1.285	0.257
	BMI (kg.m^{-2})	0.869	0.351
	IL6 (ng/l)	1.892	0.169
	CRP (mg/l)	3.219	0.073
	N/L ratio	3.268	0.071
	D-Dimer (mg/l)	4.104	0.043
	Baricitinib	0.376	0.540
Step 5	Duration of symptoms (days)	1.253	0.263
	BMI (kg.m^{-2})	0.963	0.326
	IL6 (ng/l)	1.719	0.190
	CRP (mg/l)	2.017	0.156
	N/L ratio	2.943	0.086
	Baricitinib	0.574	0.449

BMI: body mass index; CRP: C-reactive protein; IL-6: interleukin 6; n: number, N/L ratio: neutrophil-to-lymphocyte ratio; p-value: probability (univariate)

Supplementary Table S8: Cox regression model – forward stepwise method of association of in-hospital death with anti-S SARS-CoV-2 negativity and other variables. Variables in the equation of regression model.

		Beta	Beta SE	Wald	p-value	OR	Lower 95% CI	Upper 95% CI
Step 1	Age (years)	0.046	0.021	4.819	0.028	1.047	1.005	1.092
Step 2	Age (years)	0.047	0.021	4.969	0.026	1.048	1.006	1.092
	Anti-S SARS-CoV-2 negative	1.034	0.484	4.577	0.032	2.814	1.091	7.258

A forward stepwise method (probability for stepwise: entry $p < 0.05$) for Cox regression has been used to reduce the number of independent variables entering the model to reduce the probability of model overfitting.
CI: confidence interval; n: number; OR: odds ratio; p-value: probability (multivariate), SE: standard error.

Supplementary Table S9: Cox regression model – forward stepwise method of association of in-hospital death with anti-S SARS-CoV-2 negativity and other variables. Variables not in the equation of regression model.

		Score	p-value
Step 1	Moderate disease	5.460	0.065
	Severe disease	0.399	0.528
	Critical disease	2.252	0.133
	BMI ($kg.m^{-2}$)	1.988	0.159
	IL6 (ng/l)	0.127	0.722
	CRP (mg/l)	0.016	0.899
	N/L ratio	1.152	0.283
	D-Dimer (mg/l)	0.010	0.921
	Comorbidity number (n)	0.363	0.547
	Baricitnib	0.340	0.560
	Anti-S SARS-CoV-2 negative	5.000	0.025
Step 2	Moderate disease	3.879	0.144
	Severe disease	0.255	0.614
	Critical disease	1.674	0.196
	BMI ($kg.m^{-2}$)	2.573	0.109
	IL6 (ng/l)	0.492	0.483
	CRP (mg/l)	0.000	0.984
	N/L ratio	2.411	0.120
	D-Dimer (mg/l)	0.106	0.745
	Comorbidity number (n)	0.473	0.491
	Baricitnib	0.112	0.738

BMI: body mass index; CRP: C-reactive protein; IL-6: interleukin 6; n: number, N/L ratio: neutrophil-to-lymphocyte ratio; p-value: probability (univariate), SE: standard error.

Supplementary Table S10: Cox regression model – forward stepwise method of association of in-hospital death with anti-S SARS-CoV-2 IgM negativity and other variables. Variables in the equation of regression model.

		Beta	Beta SE	Wald	p - value	OR	Lower 95% CI	Upper 95% CI
Step 1	Age (years)	0.046	0.021	4.819	0.028	1.047	1.005	1.092
Step 2	Age (years)	0.045	0.020	4.838	0.028	1.046	1.005	1.089
	Anti-S SARS-CoV-2 IgM negative	1.003	0.484	4.304	0.038	2.728	1.057	7.039

A forward stepwise method (probability for stepwise: entry $p < 0.05$) for Cox regression has been used to reduce the number of independent variables entering the model to reduce the probability of model overfitting.
CI: confidence interval; IgM: immunoglobulin M, n: number, N/L ratio: neutrophil-to-lymphocyte ratio; OR: odds ratio; p-value: probability, SE: standard error.

Supplementary Table S11: Cox regression model – forward stepwise method of association of in-hospital death with anti-S SARS-CoV-2 IgM negativity and other variables. Variables not in the equation of regression model.

		Score	p-value
Step 1	Moderate disease	5,460	0,065
	Severe disease	0,399	0,528
	Critical disease	2,252	0,133
	BMI (kg.m^{-2})	1,988	0,159
	IL6 (ng/l)	0,127	0,722
	CRP (mg/l)	0,016	0,899
	N/L ratio	1,152	0,283
	D-Dimer (mg/l)	0,010	0,921
	Comorbidity number (n)	0,363	0,547
	Baricitnib	0,340	0,560
	Anti-S SARS-CoV-2 IgM negative	4,676	0,031
Step 2	Moderate disease	3,932	0,140
	Severe disease	0,223	0,637
	Critical disease	1,601	0,206
	BMI (kg.m^{-2})	2,606	0,106
	CRP (mg/l)	0,602	0,438
	IL6 (ng/l)	0,000	0,988
	N/L ratio	3,438	0,064
	D-Dimer (mg/l)	0,127	0,721
	Comorbidity number (n)	0,345	0,557
	Baricitnib	0,178	0,673

BMI: body mass index; CRP: C-reactive protein; IgM: immunoglobulin M, IL-6: interleukin 6; n: number, N/L ratio: neutrophil-to-lymphocyte ratio; p-value: probability (univariate), SE: standard error

Supplementary Table S12: Cox regression model – forward stepwise method of association of in-hospital death with anti-S SARS-CoV-2 IgG negativity and other variables. Variables in the equation of regression model.

		Beta	Beta SE	Wald	p - value	OR	Lower 95% CI	Upper 95% CI
Step 1	Age (years)	0.046	0.021	4.819	0.028	1.047	1.005	1.092
Step 2	Age (years)	0.051	0.021	5.774	0.016	1.052	1.009	1.097
	Anti-S SARS-CoV-2 IgG negative	1.215	0.569	4.561	0.033	3.369	1.105	10.270

A forward stepwise method (probability for stepwise: entry $p < 0.05$) for Cox regression has been used to reduce the number of independent variables entering the model to reduce the probability of model overfitting.
BMI: body mass index; CI: confidence interval; IgG: immunoglobulin G, n: number; OR: odds ratio; p-value: probability (multivariate), SE: standard error.

Supplementary Table S13: Cox regression model – forward stepwise method of association of in-hospital death with anti-S SARS-CoV-2 IgG negativity and other variables. Variables not in the equation of regression model.

		Score	p-value
Step 1	Moderate disease	5.460	0.065
	Severe disease	0.399	0.528
	Critical disease	2.252	0.133
	BMI ($kg.m^{-2}$)	1.988	0.159
	CRP (mg/l)	0.127	0.722
	IL6 (ng/l)	0.016	0.899
	N/L ratio	1.152	0.283
	D-Dimer (mg/l)	0.010	0.921
	Comorbidity number (n)	0.363	0.547
	Baricitnib	0.340	0.560
	Anti-S SARS-CoV-2 IgG negative	5.137	0.023
Step 2	Moderate disease	4.873	0.087
	Severe disease	0.293	0.588
	Critical disease	1.930	0.165
	BMI ($kg.m^{-2}$)	2.367	0.124
	CRP (mg/l)	0.510	0.475
	IL6 (ng/l)	0.002	0.964
	N/L ratio	2.607	0.106
	D-Dimer (mg/l)	0.167	0.683
	Comorbidity number (n)	0.305	0.581
	Baricitinib	0.003	0.959

BMI: body mass index; CRP: C-reactive protein; IgG: immunoglobulin G, IL-6: interleukin 6; n: number, N/L ratio: neutrophil-to-lymphocyte ratio; p-value: probability (univariate), SE: standard error.

Supplementary Table S14: Collinearity analysis of variables in the association with in-hospital death.

	tolerance	VIF
Disease severity	0.915	1.093
Duration of symptoms	0.995	1.005
Age (years)	0.664	1.506
BMI ($kg.m^{-2}$)	0.901	1.109
CRP (mg/l)	0.775	1.290
IL-6 (ng/l)	0.874	1.145
N/L ratio	0.723	1.383

D-Dimer (mg/l)	0.916	1.092
Comorbidity number (n)	0.705	1.418
Baricitnib	0.922	1.085
Anti-S SARS-CoV-2 negative	0.941	1.063

BMI: body mass index; CRP: C-reactive protein; IgG: immunoglobulin G, IL-6: interleukin 6; n: number, N/L ratio: neutrophil-to-lymphocyte ratio; p-value: probability (univariate), SE: standard error; VIF: variance inflation factor

Supplementary Table S15: Collinearity analysis of variables in association with death during the follow-up.

	tolerance	VIF
Disease severity	0.927	1.079
Duration of symptoms	0.984	1.016
Age (years)	0.692	1.446
BMI (kg.m^{-2})	0.903	1.108
CRP (mg/l)	0.760	1.316
IL-6 (ng/l)	0.813	1.230
N/L ratio	0.682	1.467
D-Dimer (mg/l)	0.887	1.127
Comorbidity number (n)	0.721	1.386
Baricitnib	0.917	1.091
Anti-S SARS-CoV-2 negative	0.939	1.065

BMI: body mass index; CRP: C-reactive protein; IgG: immunoglobulin G, IL-6: interleukin 6; n: number, N/L ratio: neutrophil-to-lymphocyte ratio; p-value: probability (univariate), SE: standard error; VIF: variance inflation factor.
